# A hybrid explicit implicit staggered grid finite-difference scheme for the first-order acoustic wave equation modeling

**DOI:** 10.1038/s41598-022-15112-x

**Published:** 2022-06-29

**Authors:** Wenquan Liang, Yanfei Wang, Jingjie Cao, Ursula Iturrarán-Viveros

**Affiliations:** 1 College of Resource Engineering, Longyan University, Longyan, 364000 People’s Republic of China; 2 Key Laboratory of Petroleum Resources Research, Institute of Geology and Geophysics, Chinese Academy of Sciences, Beijing, 100029 People’s Republic of China; 3 Key Laboratory of Intelligent Detection and Equipment for Underground Space of Beijing-Tianjin-Hebei Urban Agglomeration, Ministry of Natural Resources, Hebei GEO University, Shijiazhuang, 050031 People’s Republic of China; 4 Department of Mathematics, Universidad Nacional Autónoma de México, Mexico City, Mexico

**Keywords:** Geophysics, Applied mathematics

## Abstract

Implicit staggered-grid finite-difference (SGFD) methods are widely used for the first-order acoustic wave-equation modeling. The identical implicit SGFD operator is commonly used for all of the first-order spatial derivatives in the first-order acoustic wave-equation. In this paper, we propose a hybrid explicit implicit SGFD (HEI-SGFD) scheme which could simultaneously preserve the wave-equation simulation accuracy and increase the wave-equation simulation speed. We use a second-order explicit SGFD operator for half of the first-order spatial derivatives in the first-order acoustic wave-equation. At the same time, we use the implicit SGFD operator with added points in the diagonal direction for the other first-order spatial derivatives in the first-order acoustic wave-equation. The proposed HEI-SGFD scheme nearly doubles the wave-equation simulation speed compared to the implicit SGFD schemes. In essence, the proposed HEI-SGFD scheme is equivalent to the second-order FD scheme with ordinary grid format. We then determine the HEI-SGFD coefficients in the time–space domain by minimizing the phase velocity error using the least-squares method. Finally, the effectiveness of the proposed method is demonstrated by dispersion analysis and numerical simulations.

## Introduction

The seismic wave equations have many applications such as seismic exploration, source localization among others^1–4^. Finite difference time domain (FDTD) method is one of the most popular methods for solving the seismic wave equations ^5–7^. Staggered-grid finite-difference (SGFD) methods are part of the FDTD methods that are commonly utilized in seismic wave extrapolation because of their high accuracy, less memory requirement and easy implementation^8–11^.

To improve the accuracy and efficiency of the FD method, the FD stencil with added points in the diagonal direction was adopted^12–14^. Compared to the previous high-order FD stencils, the use of these new FD stencils could improve the wave-equation simulation efficiency while preserve the high accuracy by using a larger time step. Both the explicit and the implicit SGFD schemes are widely used for wave-equation modeling. The implicit SGFD scheme could achieve higher accuracy with much shorter FD operator than the explicit SGFD scheme^15,16^. Another way to improve the accuracy and efficiency of the FDTD method is utilizing optimized FD operator coefficients^17–21^.

Geophysical imaging needs a large amount of of wave equation modeling^22^. It usually takes a long time to perform geophysical imaging. These computations have to be deployed on supercomputers or relying on GPUs (Graphics Processing Units) in order to get the result with a tolerable waiting time^23,24^. Using efficient wave equation modeling algorithms can reduce the running time, so as to save energy and potentially reduce carbon emissions.

The identical SGFD operator is the most common operator used for all the first-order spatial derivatives in the first-order acoustic wave-equation. We suggest using unalike SGFD operators for different spatial derivatives in the acoustic wave equation ^25,26^ and propose a HEI-SGFD scheme in this paper. We use the second-order explicit SGFD scheme for some of the first-order spatial derivatives in the first-order acoustic wave-equation. Meanwhile we use the high-order implicit SGFD scheme with added points in the diagonal direction for the rest of the first-order spatial derivatives. The proposed FD scheme is called: the HEI-SGFD scheme. This HEI-SGFD scheme could save about 45 percent of the computational time compared with the Implicit SGFD scheme while it still preserves high accuracy.

## Theory

The first-order velocity-stress acoustic wave-equation can be described as follows:

1 $$\frac{\partial p}{{\partial t}} = - \rho v^{2} \left( {\frac{{\partial V_{z} }}{\partial z} + \frac{{\partial V_{x} }}{\partial x}} \right),$$

2 $$\frac{{\partial V_{x} }}{\partial t} = - \frac{1}{\rho }\frac{\partial p}{{\partial x}},$$

3 $$\frac{{\partial V_{z} }}{\partial t} = - \frac{1}{\rho }\frac{\partial p}{{\partial z}},$$

where* p* is the pressure, *ρ *is the density and *v* is the wave propagation speed, *V*_*x*_ and *V*_*z*_ are the particle velocities. A constant density is assumed for the equations in this paper.

The acoustic wave-equations (1) to (3) can be discretized as

4 $$p_{{i,j}}^{k} = p_{{i,j}}^{{k - 1}} - v^{2} \tau \left( {\Delta _{z} V_{{z(i + 1/2,j)}}^{{k + 1/2}} + \Delta _{x} V_{{x(i,j + 1/2)}}^{{k + 1/2}} } \right),$$

5 $$V_{{x(i,j + 1/2)}}^{{k + 1/2}} = V_{{x(i,j + 1/2)}}^{{k - 1/2}} - \tau \left( {\Delta _{x} p_{{i,j}}^{k} } \right),$$

6 $$V_{{z(i + 1/2,j)}}^{{k + 1/2}} = V_{{z(i + 1/2,j)}}^{{k - 1/2}} - \tau \left( {\Delta _{z} p_{{i,j}}^{k} } \right),$$

where $\tau$ is the time step, $\varsigma_{m,j}^{n} = \varsigma (z + mh,x + jh,t + n\tau ), \, \varsigma = [V_{z} ,V_{x} ,p]$. The symbol Δ represents the implicit discrete form of the spatial derivative, for example we use it in the following equation:

7 $$1 + bh^{2} \frac{{\delta^{2} \left( {\Delta_{x} p_{i,j + 1/2}^{k} } \right)}}{{\delta x^{2} }} = \frac{1}{h}\sum\limits_{m = 1}^{M} {c_{m} \left( {p_{i,j + m}^{k} - p_{i,j - m + 1}^{k} } \right) + \frac{1}{h}c_{M + 1} \left( {p_{i + 1,j + 1}^{k} - p_{i + 1,j}^{k} + p_{i - 1,j + 1}^{k} - p_{i - 1,j}^{k} } \right)} ,$$

where

8 $$\frac{{\delta^{2} q}}{{\delta x^{2} }} = \frac{q(x + h) + q(x - h) - 2q(x)}{{h^{2} }}.$$

In order to use a larger time and space grid intervals, in Eq. (8), *M* is the length of the SGFD operators, *c*_*m*_ and *b* are the SGFD coefficients and *h* is the grid size, *M* = 3 and $c_{M + 1} \ne {0}$ are adopted in this paper. When *b* and *c*_*M*+*1*_ equal zero, Eqs. (4) to (8) are conventional explicit high order SGFD scheme.

Then we can implicitly get an approximation to the spatial derivative ${{q \approx \partial p} \mathord{\left/ {\vphantom {{q \approx \partial p} {\partial x}}} \right. \kern-\nulldelimiterspace} {\partial x}}$ as

9 $$bq_{i,j + 3/2}^{k} + (1 - 2b)q_{i,j + 1/2}^{k} + bq_{i,j - 1/2}^{k} = \frac{1}{h}\left( {\sum\limits_{m = 1}^{M} {c_{m} (p_{i,j + m}^{k} - p_{i,j - m + 1}^{k} ) + c_{M + 1} (p_{i + 1,j + 1}^{k} - p_{{i{ + }1,j}}^{k} + p_{i - 1,j + 1}^{k} - p_{i - 1,j}^{k} )} } \right).$$

Hereafter, we denote Eqs. (4) to (6) as the implicit SGFD scheme. One can use a larger time step with the coefficient *c*_*M*+1_. At the same time, one can use a short operator length with the coefficient *b*^27–30^. We propose to use the simplest explicit second-order SGFD operator for the spatial derivatives in Eqs. (2) and (3). The proposed HEI-SGFD scheme is given by:

10 $$p_{{i,j}}^{k} = p_{{i,j}}^{{k - 1}} - v^{2} \tau \left( {\Delta _{z} V_{{z(i + 1/2,j)}}^{{k + 1/2}} + \Delta _{x} V_{{x(i,j + 1/2)}}^{{k + 1/2}} } \right),$$

11 $$V_{{x(i,j + 1/2)}}^{{k + 1/2}} = V_{{x(i,j + 1/2)}}^{{k - 1/2}} - \frac{\tau }{h}\left( {p_{{i,j + 1}}^{k} - p_{{i,j}}^{k} } \right),$$

12 $$V_{{z(i + 1/2,j)}}^{{k + 1/2}} = V_{{z(i + 1/2,j)}}^{{k - 1/2}} - \frac{\tau }{h}\left( {p_{{i + 1,j}}^{k} - p_{{i,j}}^{k} } \right).$$

The SGFD scheme in Eq. (10) is implicit while the SGFD schemes in Eqs. (11) and (12) are explicit and second-order. Therefore, we refer the proposed SGFD scheme as the HEI-SGFD scheme. The HEI-SGFD scheme is an implicit SGFD scheme although some of spatial derivatives are approximated with explicit SGFD scheme. It seems that Eqs. (11) and (12) are not accurate, nevertheless, it is not the case. The SGFD coefficient in Eq. (10) is determined by considering Eqs. (11) and (12). It is easily observed that the proposed HEI-SGFD scheme in Eqs. (10) to (12) needs less floating-point computations compared with the implicit SGFD scheme given in Eqs. (4)–(6). The computational resources needed by Eqs. (11) and (12) are smaller than that of Eqs. (5) and (6).

Substitute Eqs. (11) and (12) into Eq. (10) and we get:

13 $${{p}}_{{{i,j}}}^{{{{k}}{ + 1}}} \user2{ = }{2}{{p}}_{{{i,j}}}^{{{k}}} - {{p}}_{{{i,j}}}^{{{{k}} - {{1}}}} + \frac{{{{v}}^{{{2}}} {{\tau}}^{{2}} }}{{{{h}}^{{2}} }}p_{{(i,j)_{z} }}^{{k^{{\prime \prime }} }} + \frac{{{v}^{{2}} {\tau }^{{\text{2}}} }}{{{h}^{{\text{2}}} }}p_{{(i,j)_{x} }}^{{k^{{\prime \prime }} }},$$

where

14 $$1 + bh^{2} \frac{{\delta ^{2} \left( {p_{{(i,j)_{z} }} ^{{\prime \prime }} } \right)}}{{\delta z^{2} }} = - 2c_{1} p_{{i,j}}^{k} + \sum\limits_{{m = 1}}^{{M - 1}} {\left( {c_{m} - c_{{m + 1}} } \right)(p_{{i + m,j}}^{k} + p_{{i - m,j}}^{k} ) + c_{M} (p_{{i + M,j}}^{k} + p_{{i - M,j}}^{k} ) + c_{{M + 1}} (p_{{i + 1,j + 1}}^{k} + p_{{i - 1,j + 1}}^{k} + p_{{i + 1,j - 1}}^{k} + p_{{i - 1,j - 1}}^{k} )} ,\user2{ }$$

15 $$1 + bh^{2} \frac{{\delta ^{2} \left( {p_{{(i,j)_{x} }} ^{{\prime \prime }} } \right)}}{{\delta x^{2} }} = \user2{ } - 2c_{1} p_{{i,j}}^{k} + \sum\limits_{{m = 1}}^{{M - 1}} {\left( {c_{m} - c_{{m + 1}} } \right)(p_{{i,j + m}}^{k} + p_{{i,j - m}}^{k} ) + c_{M} (p_{{i,j + M}}^{k} + p_{{i,j - M}}^{k} ) + c_{{M + 1}} (p_{{i + 1,j + 1}}^{k} + p_{{i + 1,j - 1}}^{k} + p_{{i - 1,j + 1}}^{k} + p_{{i - 1,j - 1}}^{k} )} .$$

In theory, the first-order HEI-SGFD scheme in Eqs. (10) to (12) is equivalent to the second-order implicit FD scheme with ordinary grid format as shown in Eq. (13).

The dispersion relation of the proposed HEI-SGFD scheme is given by

16 $$\left[ { - 2\sin \left( {0.5k_{x} h} \right)\sum\limits_{m = 1}^{M} {2c_{m} } \sin \left( {(m - 0.5)k_{x} h} \right) + 4c_{M + 1} \cos (k_{z} h)(\cos (k_{x} h) - 1)} \right]\left[ {{1 + 2}b{\text{(cos(}}k_{z} h{)} - {1)}} \right] + \left[ { - 2\sin \left( {0.5k_{z} h} \right)\sum\limits_{m = 1}^{M} {2c_{m} } \sin \left( {(m - 0.5)k_{z} h} \right) + 4c_{M + 1} \cos (k_{x} h)(\cos (k_{z} h) - 1)} \right]\left[ {{1 + 2}b{\text{(cos(}}k_{x} h{)} - {1)}} \right] = \frac{1}{{r^{2} }}\left[ {{1 + 2}b{\text{(cos(}}k_{x} h{)} - {1)}} \right]\left[ {{1 + 2}b{\text{(cos(}}k_{z} h{)} - {1)}} \right]\left[ {2\cos \left( {kv\tau } \right) - 2} \right].$$

The SGFD coefficients in Eq. (16) should be carefully determined with the optimization methods. Similar to the method proposed by Wang et al.^31^, we get the objective function from Eq. (16) by minimizing the error between the true velocity and the phase velocity:

17 $$\Phi (c) = \sum\limits_{{k = {\text{3e - 12}}}}^{K} {\sum\limits_{\theta = 0}^{\pi /4} {\left( {\frac{{v_{fd} }}{v} - 1} \right)^{2} } } = \sum\limits_{{k = {\text{ 3e - 12}}}}^{K} {\sum\limits_{\theta = 0}^{\pi /4} {\left( {\frac{{a{\text{cos}}\left( {1 + \frac{{r^{2} g}}{{2\left[ {1 + 2b\left( {\cos \left( {k_{z} h} \right) - 1} \right)} \right]\left[ {1 + 2b\left( {\cos \left( {k_{x} h} \right) - 1} \right)} \right]}}} \right)}}{kv\tau } - 1} \right)^{2} } } ,$$

where

18 $$\begin{aligned} \, g & = \, \left[ { - 2\sin \left( {0.5k_{x} h} \right)\sum\limits_{m = 1}^{M} {2c_{m} } \sin \left( {(m - 0.5)k_{x} h} \right) + 4c_{M + 1} \cos (k_{z} h)(\cos (k_{x} h) - 1)} \right]\left[ {{1 + 2}b{\text{(cos(}}k_{z} h{)} - {1)}} \right] \\ & \quad + \left[ { - 2\sin \left( {0.5k_{z} h} \right)\sum\limits_{m = 1}^{M} {2c_{m} } \sin \left( {(m - 0.5)k_{z} h} \right) + 4c_{M + 1} \cos (k_{x} h)(\cos (k_{z} h) - 1)} \right]\left[ {{1 + 2}b{\text{(cos(}}k_{x} h{)} - {1)}} \right]. \\ \end{aligned}$$

The only unknowns in Eq. (17) are $c_{m}$ ($m = 1, \cdots ,M + 1$) and *b*. The wave number *k* in Eq. (17) should start from zero. However, *k* appears in the denominator, which causes instability. Therefore, we let *k* start from a very small number, e.g.,$3.0 \times 10^{ - 12}$. We assume that the parameters such as the wave propagation speed, the time step and the spatial grid intervals are already given (then *r* = $v\tau$/*h* will be known). The other two parameters are *k* and *θ*. The propagation angle *θ* is from 0 to *π*/4 (0, *π*/16 , 2*π*/16 … *π*/4). From Eq. (17), we found that in the frequency-wave number domain the SGFD coefficients are related to the Courant ratio *r*. When one considers a wave-equation simulation with both fixed time and spatial steps grid intervals, the SGFD coefficient is different for different velocities and the stencil forms a big 3D matrix for a 2D complex velocity model. In order to get the hybrid explicit-implicit SGFD coefficient we apply the MATLAB function *lsqnonlin* to solve the nonlinear least-squares problem in Eq. (17).

The proposed HEI-SGFD scheme can be easily extended to 3D:

19 $$p_{{l,m,n}}^{k} = p_{{l,m,n}}^{{k - 1}} - v^{2} \tau \left( {\Delta _{z} V_{{z(l+1/2,m,n)}}^{{k + 1/2}} + \Delta _{x} V_{{x(l,m+1/2,n)}}^{{k + 1/2}} + \Delta _{y} V_{{y(l,m,n+1/2)}}^{{k + 1/2}} } \right),$$

20 $$V_{{x(l,m + 1/2,n)}}^{{k + 1/2}} = V_{{x(l,m + 1/2,n)}}^{{k - 1/2}} - \frac{\tau }{h}\left( {p_{{l,m + 1,n}}^{k} - p_{{l,m,n}}^{k} } \right),$$

21 $$V_{{y(l,m , n + 1/2)}}^{{k + 1/2}} = V_{{y(l,m, n + 1/2)}}^{{k - 1/2}} - \frac{\tau }{h}\left( {p_{{l,m,n + 1}}^{k} - p_{{l,m,n}}^{k} } \right),$$

22 $$V_{{z(l + 1/2,m,n)}}^{{k + 1/2}} = V_{{z(l + 1/2,m,n)}}^{{k - 1/2}} - \frac{\tau }{h}\left( {p_{{l + 1,m,n}}^{k} - p_{{l,m,n}}^{k} } \right),$$

where $\tau$ is the time step, $\varsigma_{l,m,n}^{k} = \varsigma (z + lh,x + mh,y + nh,t + k\tau ), \, \varsigma = [V_{z} ,V_{x} ,V_{y} ,p].$ The SGFD coefficient in Eq. (19) of the hybrid explicit-implicit SGFD scheme for 3D can be determined similarly.

## Dispersion analysis and stability analysis

In the following, we will demonstrate that the proposed HEI-SGFD scheme possesses similar accuracy compared to the computational-intensive traditional implicit SGFD scheme.

The 2D dispersion error of the HEI-SGFD scheme is defined as follows.

23 $$\delta = \frac{{v_{FD} }}{v} = \frac{1}{krh}a\cos \left( {1 + \frac{{r^{2} }}{2}\frac{g}{{\left[ {1 + 2b(\cos (k_{x} h) - 1)} \right]\left[ {1 + 2b(\cos (k_{z} h) - 1)} \right]}}} \right).$$

The difference between the FD propagation time and the exact propagation time through one grid is defined as

24 $$\varepsilon = \frac{h}{{v_{FD} }} - \frac{h}{v} = \frac{h}{v}\left( {\frac{v}{{v_{FD} }} - 1} \right) = \frac{h}{v}\left( {\frac{1}{\delta } - 1} \right).$$

**Figure 1:**
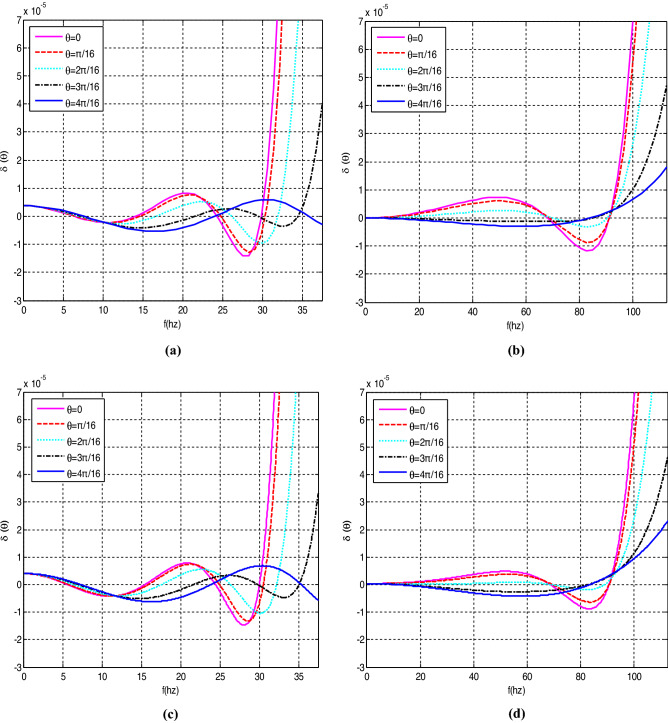
Dispersion error curves for the different implicit SGFD schemes. The time step is 2.5 ms and the spatial grid interval is 20 m. (**a**) The implicit SGFD scheme with v = 1500 m/s; (**b**) the implicit SGFD scheme with v = 4500 m/s; (**c**) the HEI-SGFD scheme with v = 4500 m/s; (**d**) the HEI-SGFD scheme with v = 4500 m/s.

Figure 1 shows the dispersion error curves for the implicit SGFD scheme and the HEI-SGFD scheme with the SGFD coefficient optimized in the time–space domain for different velocities. The spatial grid interval is 20 m and the time step is 2.5 ms. The velocities are 1500 m/s for Fig. 1a,c, and 4500 m/s for Fig. 1b,d, respectively. The implicit SGFD coefficients used in Fig. 1 are shown in Table 1. We observed that the implicit SGFD method can preserve the dispersion relation at a large frequency range even when *M* (which characterizes the width of the stencil) is equal to 3. The grid dispersion in Fig. 1c,d is similar to the one in Fig. 1a,b. However, the wave-equation simulation speed could be nearly doubled with the HEI-SGFD scheme.

**Table 1:** The implicit SGFD coefficients used to obtain the dispersion error curves in Fig. 1.

*v*	*c*_1_	*c*_2_	*c*_3_	*c*_4_	*b*	*r*
1500	0.491536	0.176179	− 0.00451725	0.00111733	0.195121	0.1875
4500	0.499944	0.162557	− 0.00178656	0.0106658	0.180653	0.5625
1500	0.55651	0.159946	− 0.00839528	0.00251221	0.201776	0.1875
4500	0.506957	0.156756	− 0.00429556	0.0220486	0.191268	0.5625

The first and second rows are the implicit SGFD coefficients used for Fig. 1a,b. The third and fourth rows are the HEI-SGFD coefficients used for Fig. 1c,d.

When applying the Fourier transforms to the SGFD operators for the spatial derivatives in Eq. (7), we get

25 $$k_{x} \approx \frac{{ - \left( {\sum\nolimits_{m = 1}^{M} {2c_{m} } \sin \left( {(m - 0.5)k_{x} h} \right) + {4}c_{M + 1} \sin (k_{x} h/2)\cos (k_{z} h)} \right)}}{{h\left( {{1 + 2}b{\text{(cos(}}k_{x} h{)} - {1)}} \right)}}.$$

Similarly, we can get

26 $$k_{z} \approx \frac{{ - \left( {\sum\nolimits_{m = 1}^{M} {2c_{m} } \sin \left( {(m - 0.5)k_{z} h} \right) + {4}c_{M + 1} \sin (k_{z} h/2)\cos (k_{x} h)} \right)}}{{h\left( {{1 + 2}b{\text{(cos(}}k_{z} h{)} - {1)}} \right)}}.$$

Then we can get the dispersion relation for the implicit SGFD scheme

27 $$- \left[ {\sum\limits_{m = 1}^{M} {2c_{m} } \sin \left( {(m - 0.5)k_{x} h} \right) + {4}c_{M + 1} \sin (k_{x} h/2)\cos (k_{z} h)} \right]^{2} \left[ {{1 + 2}b{\text{(cos(}}k_{z} h{)} - {1)}} \right]^{{2}} - \left[ {\sum\limits_{m = 1}^{M} {2c_{m} } \sin \left( {(m - 0.5)k_{z} h} \right) + {4}c_{M + 1} \sin (k_{z} h/2)\cos (k_{x} h)} \right]^{2} \left[ {{1 + 2}b{\text{(cos(}}k_{x} h{)} - {1)}} \right]^{{2}} = \frac{1}{{r^{2} }}\left[ {{1 + 2}b{\text{(cos(}}k_{x} h{)} - {1)}} \right]^{{2}} \left[ {{1 + 2}b{\text{(cos(}}k_{z} h{)} - {1)}} \right]^{{2}} \left[ {2\cos \left( {kv\tau } \right) - 2} \right].$$

From Eq. (27) we get the CFL condition for the implicit SGFD scheme

28 $$r \le s = \sqrt {\frac{{2\left[ {{1} - {4}b} \right]^{{2}} }}{{\left[ {\sum\nolimits_{m = 1}^{M} {2c_{m} } ( - 1)^{m - 1} - {4}c_{M + 1} } \right]^{2} }}}.$$

From dispersion relation Eq. (16), we can get

29 $$\, \frac{{r^{2} g}}{{\left[ {{1 + 2}b{\text{(cos(}}k_{x} h{)} - {1)}} \right]\left[ {{1 + 2}b{\text{(cos(}}k_{z} h{)} - {1)}} \right]}} \ge - 4.$$

Then the stability condition of the HEI-SGFD scheme can be obtained as follows

30 $$r \le s = \sqrt {\frac{{{1 - 4}b}}{{\sum\nolimits_{m = 1}^{M} {2c_{m} } ( - 1)^{m - 1} - 4c_{M + 1} }}}.$$

**Figure 2:**
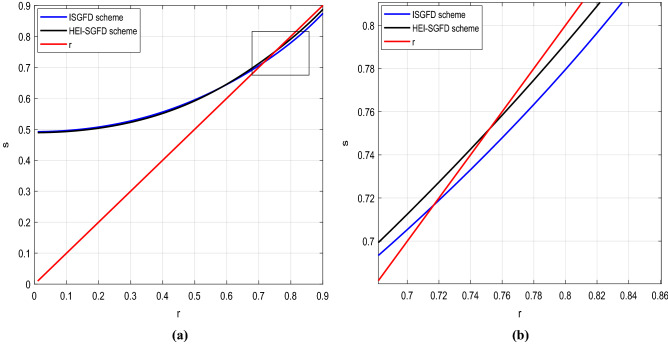
Comparison of the stability condition. (**a**) Stability condition of different implicit SGFD schemes; (**b**) local enlargement of (**a**) in the rectangle.

Figure 2 illustrates the stability condition of the implicit SGFD scheme and the HEI-SGFD scheme. The stability condition of the implicit SGFD scheme is that *r* is less than 0.715. The stability condition of the HEI-SGFD scheme is that *r* is less than 0.75. This means that the HEI-SGFD scheme has a better stability condition than the implicit SGFD scheme. We use the wave-equation simulation to verify the soundness of the stability condition. At first, we set *h* = 10 m, *v* = 7400 m/s and $\tau = 1$ ms (so that *r* = 0.74). It yields that for the implicit SGFD scheme, it is unstable. Nevertheless, the HEI-SGFD scheme is stable. This further indicates that the HEI-SGFD scheme has a better stability condition than the traditional implicit SGFD scheme.

## Examples

We first consider a homogeneous model. The seismic source position is at the center of the model. The wave propagation speed is 1500 m/s and the spatial grid interval is 0.25 m. The time step is 0.1 ms for the SGFD methods. A Ricker wavelet with a main frequency at 300 Hz is used as seismic source. The *analytical* function provided by Devito is used to get the reference solution^32^.

**Figure 3:**
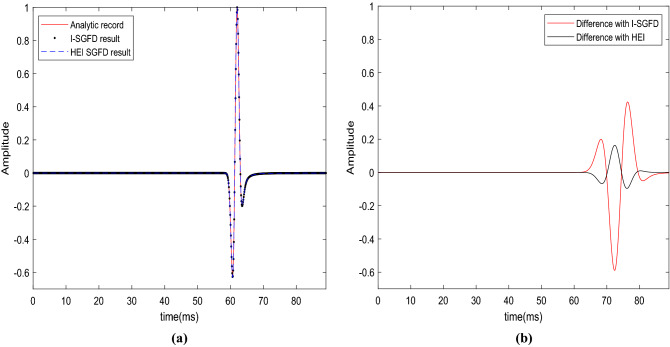
Accuracy comparison between the SGFD result and the analytical result. (**a**) The SGFD results overlapped with the analytical result; (**b**) 100 × (the difference between the SGFD results and the analytical result).

The seismograms obtained by 2 different implicit SGFD schemes are presented in Fig. 3. Both of the SGFD coefficients in Fig. 3 are determined in the time–space domain by the least-squares method. Table 2 shows the SGFD coefficients for the implicit SGFD scheme and the HEI-SGFD scheme.

**Table 2:** The SGFD coefficients used to obtain the seismograms in Fig. 3.

*c*_1_	*c*_2_	*c*_3_	*c*_4_	*b*
0.528057	0.150694	− 0.00114638	0.0128301	0.170061
0.513117	0.150794	− 0.00330739	0.0255127	0.185831

The first row is the implicit SGFD coefficients used for Eqs. (4) to (6), the second row is the HEI-SGFD coefficients used for Eq. (10).

From Fig. 3a,b, we can observe that the seismograms obtained by the implicit SGFD scheme and the HEI-SGFD scheme are almost identical to each other. However, the cost of computation of the HEI-SGFD scheme is about 55 percent of the implicit SGFD scheme. In our simulation, there are 880 grid points in the x and the z directions. With the implicit SGFD scheme, the simulation time is 307 s. With the proposed HEI-SGFD grid scheme, the simulation time is 171 s. The snapshots of the *Vz* and *Vx* components show similar patterns, and we provide the source codes to reproduce the results for the interested readers.

**Figure 4:**
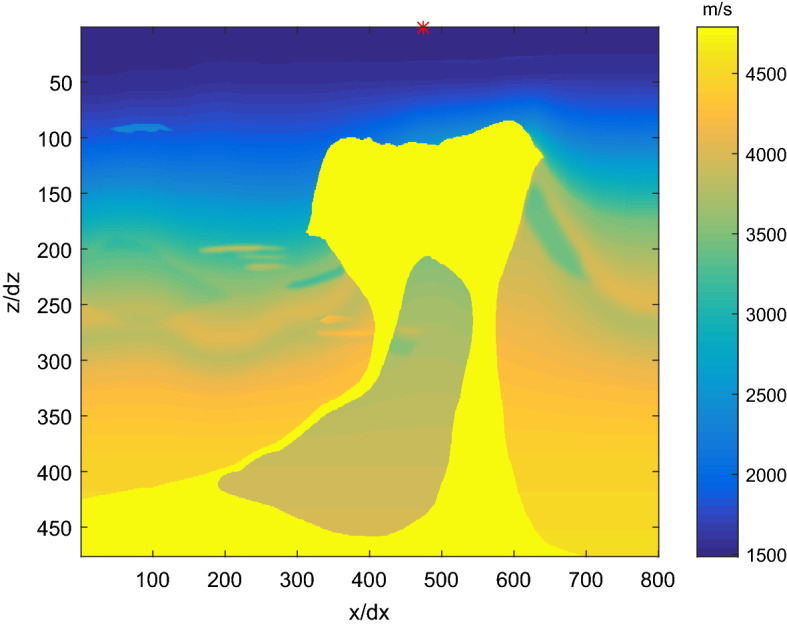
The BP salt velocity model.

Figure 4 shows the commonly used BP salt velocity model in geophysical community^33^. There are 475 grid points in the z direction and 799 grid points in the x direction (this does not include the grid nodes for the absorbing boundary). Boundary condition is also an important research area in wave equation modeling^34^. The *spongeABC* function provided by the CREWES Project with 45 absorbing grid points at four sides was used as the boundary condition^34–36^.

The seismic source position is shown as a red star. The spatial sampling interval is 15 m. All FD methods used the temporal step of 2 ms. A Ricker wavelet with a dominant frequency at 14.3 Hz was used as the seismic source.

**Figure 5:**
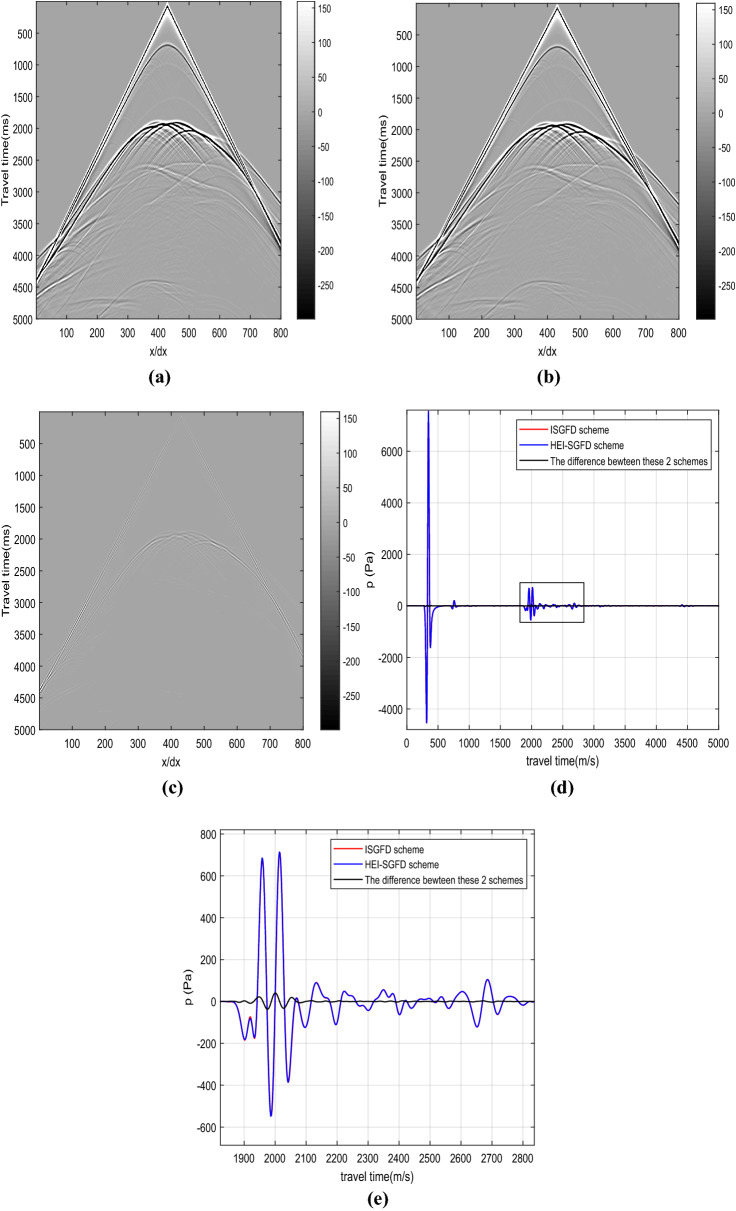
Seismic records *p* obtained with different SGFD schemes. (**a**) The implicit SGFD scheme; (**b**) the HEI-SGFD scheme; (**c**) the difference between (**a**) and (**b**); (**d**) the seismograms from (**a**) to (**b**) at x/dx = 405; (**e**) local enlargement of (**d**).

Figure 5 shows the seismic records and seismograms for the *p* component obtained by the implicit SGFD scheme and the proposed HEI-SGFD scheme. From Fig. 5a–c, it is observed that the seismic records are similar to each other. From Fig. 5d,e, it can be further observed that the seismograms are overlapped to each other for the two methods. However, with the proposed HEI-SGFD scheme, the simulation time is reduced by almost 45 percent. For example, in our simulation, with the implicit SGFD scheme, the simulation time is 325 s; while with the proposed HEI-SGFD grid scheme, the simulation time is 179 s. The significant reduction of simulation time is mainly the result of using the simplest explicit SGFD operator in Eqs. (11) and (12).

## Conclusion

In this paper, we bring forward a HEI-SGFD scheme for the first-order acoustic wave-equation modeling. The proposed HEI-SGFD scheme possesses two major advantages compared to the conventional implicit SGFD schemes. The first one is that the length of the SGFD operator in Eqs. (11) and (12) is much shorter than that of the SGFD operator in Eqs. (5) and (6). The second advantage is that the SGFD operator in Eqs. (11) and (12) are explicit. Through dispersion analysis and numerical simulation, we conclude that the proposed HEI-SGFD scheme is more efficient than the conventional implicit SGFD scheme while simultaneously preserving high accuracy. As a result, the HEI-SGFD scheme could be widely adopted for first-order acoustic wave-equation simulations.

## Data Availability

The datasets used and/or analyzed during the current study available from the corresponding author on reasonable request.
